# Supplementary material for: Neuropilin 1 and its inhibitory ligand mini-tryptophanyl-tRNA synthetase inversely regulate VE-cadherin turnover and vascular permeability
Source: Nat Commun. 2022 Jul 20;13:4188. doi: 10.1038/s41467-022-31904-1 (PMC9300702; doi:10.1038/s41467-022-31904-1)
Supplement: Supplementary file 1 — Supplementary Information [file 41467_2022_31904_MOESM1_ESM.pdf]

SUPPLEMENTARY INFORMATION

Neuropilin 1 and its inhibitory ligand mini-tryptophanyl-tRNA synthetase inversely regulate VE-cadherin turnover and vascular permeability

Noemi Gioelli, Lisa J. Neilson, Na Wei, Giulia Villari, Wenqian Chen, Bernhard Kuhle, Manuel Ehling, Federica Maione, Sander Willox, Serena Brundu, Daniele Avanzato, Grigorios Koulouras, Massimiliano Mazzone, Enrico Giraudo, Xiang-Lei Yang, Donatella Valdembri, Sara Zanivan, and Guido Serini

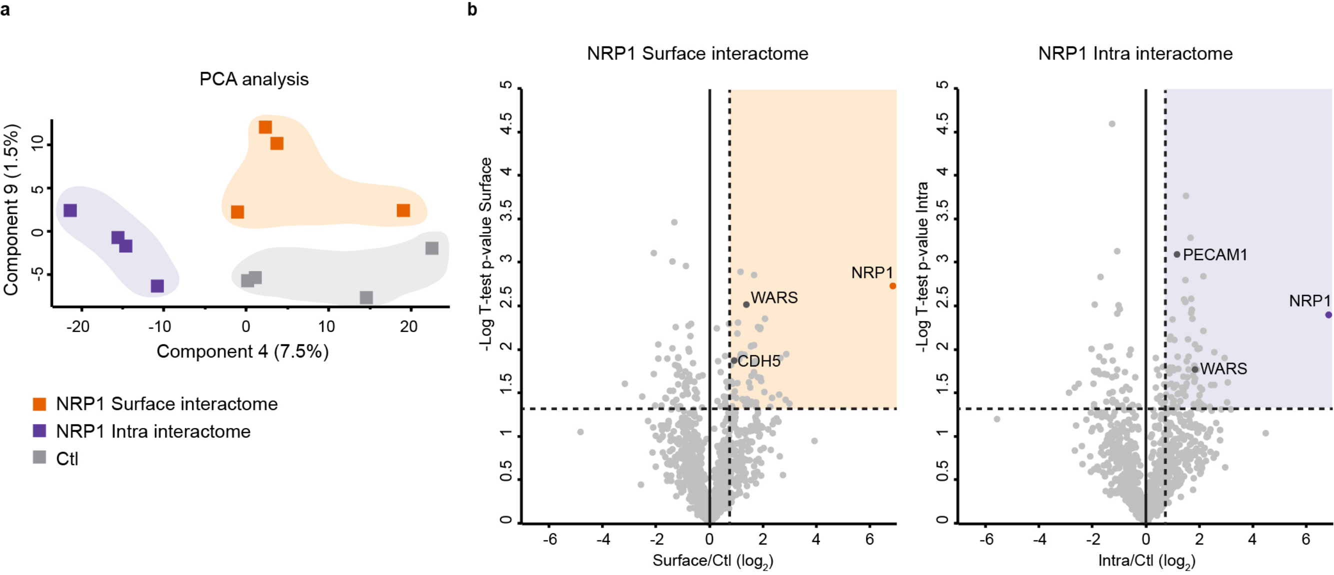

Supplementary Figure 1. Analysis of NRP1 interactome. (a) PCA Two principal components, which captured 9% of the total variance of the NRP1 interactome in control samples, NRP1 surface interactome and NRP1 intra interactome, are shown. Plots were generated with the Perseus software and edited in Adobe Illustrator. (b) VE-cadherin (CDH5), PECAM-1, and WARS are potential NRP1 binding partners. Volcano plots showing the results of the single sample t-test performed on the ratios calculated between NRP1 surface interactome (*left*) or NRP1 intra interactome (*right*) with the control sample. The dashed lines highlight the minimum ratio (NPR1 interactome/Ctrl) of 1.5-fold (0.058 in $\log_2$ scale, x axis) and p-value of 0.05 (1.3 in –Log scale, y axis), which were used to select NRP1 potential binding partners. The names of the proteins for which follow up experiments were performed are highlighted.

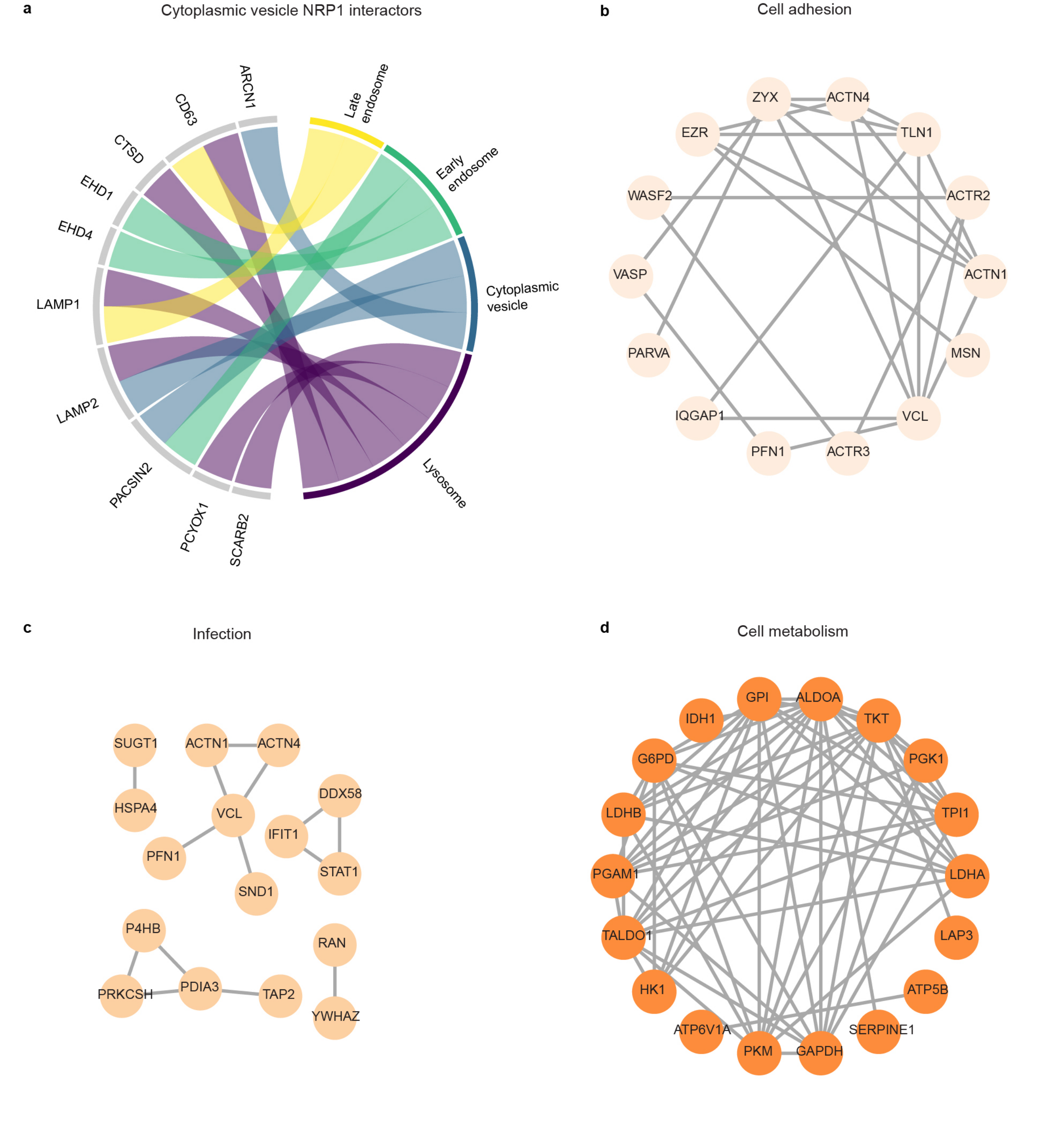

Supplementary Figure 2. Pathway enrichment of NRP1 interactome. (a) Chord diagram of proteins found to interact with NRP1 and that localize to one or more of the indicated endosomal compartment. Each protein was assigned to one or more subcellular locations based on UniProt annotation. (b-d) After excluding transmembrane (Fig. 2b) and endosomal (a) NRP1 interactors. STRING was used to perform enrichment analysis of KEGG pathways of NRP1 interactors. In addition to the KEGG category of aminoacyl-tRNA biosynthesis (Fig. 2c), proteins belonging to the KEGG categories identified with STRING were grouped in three additional main categories: cell adhesion (b), infection (c), and cell metabolism (d). Then, we built up an interaction map based on the physical and functional interactions of the proteins belonging to those three categories. The network of proteins related to infection (c) contained proteins involved in MHC class I antigen presentation (antigen peptide transporter TAP2) and the transcription activator STAT1, which mediates cellular responses to pro-inflammatory cytokines, such as interferons (IFNs). The cell metabolism network (d) contained instead many enzymes of glucose metabolism, including those of the glycolytic pathway. Plots in panels b-d were generated with R.

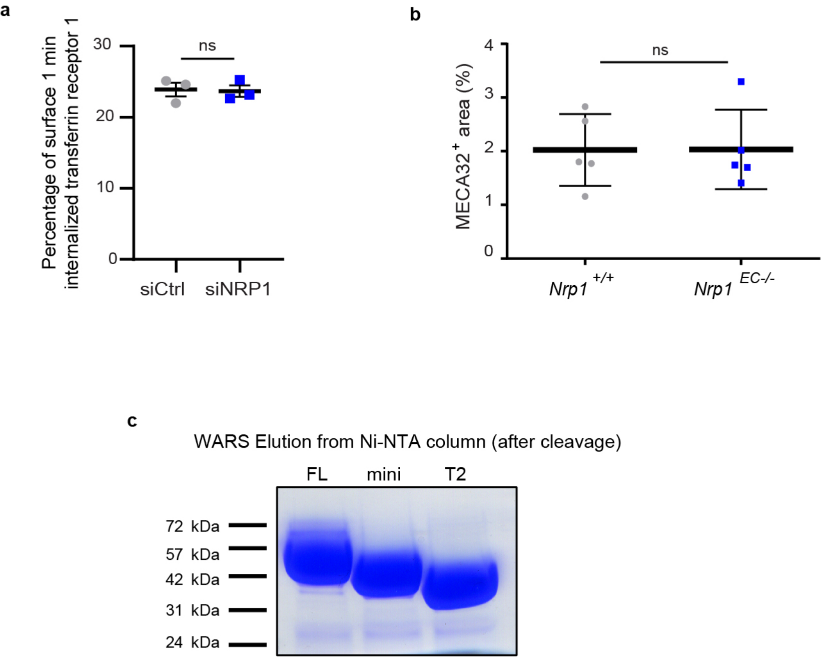

Supplementary Figure 3. Nrp1 silencing effect on transferrin receptor 1 endocytosis in vitro and blood vessel density in vivo and WARS protein production. (a) Percentage of transferrin receptor 1 endocytosis in siCtrl or siNRP1 ECs measured by capture ELISA. Data are the mean of three independent experiments ± SEM. One-way ANOVA with Bonferroni's post hoc analysis; ns = p-value ≥ 0.05. (b) Blood vessel density in either *Nrp1*$^{+/+}$ or *Nrp1*$^{EC-/-}$ mice was evaluated as MECA32^{+} percentage area. Results are the mean ± SD (n = 5 per group). Two-tailed heteroscedastic Student's t-test; ns = p-value ≥ 0.05. (c) Purified recombinant WARS proteins. A representative experiment out of three is shown.

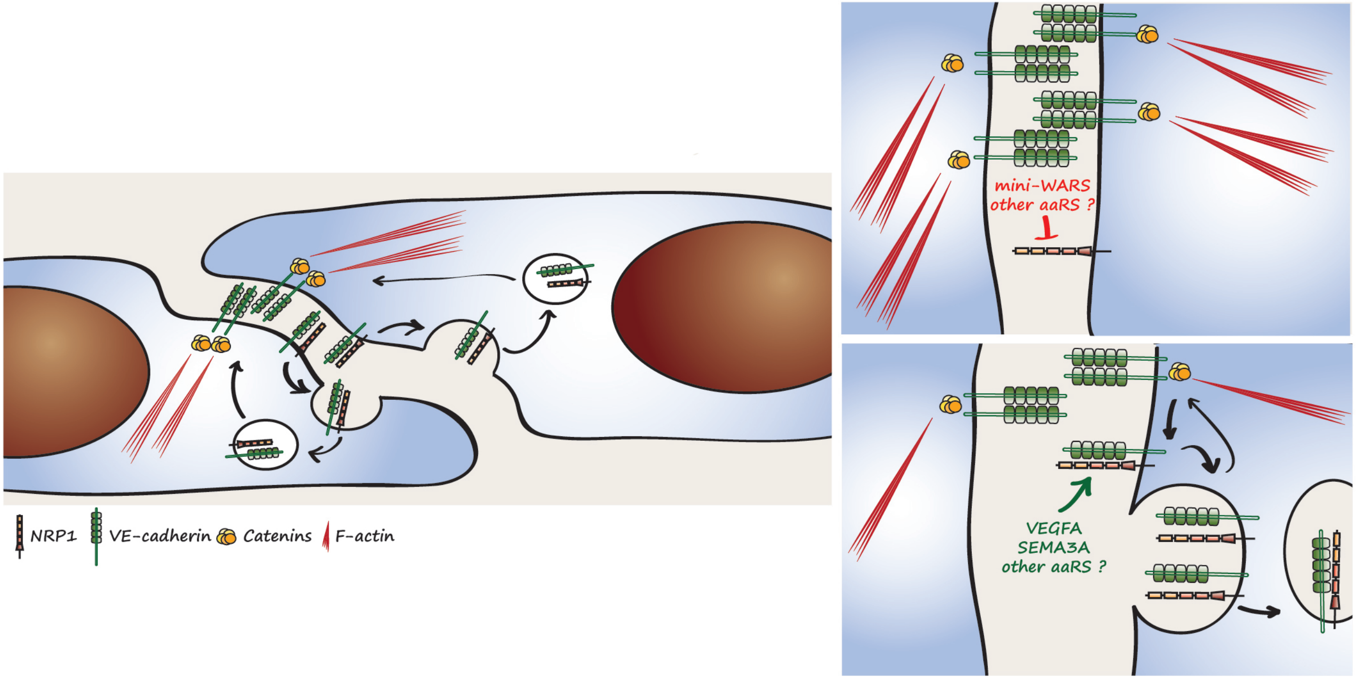

Supplementary Figure 4. Activatory and inhibitory ligands modulate NRP1 pro-endocytic activity on VE-cadherin turnover at endothelial AJs. Left panel, At endothelial AJs, NRP1 interacts with cell-to-cell adhesion receptor VE-cadherin, promoting its internalization and turnover, a function known to be crucial for both blood vessel formation and vascular homeostasis. Right panels, Inhibiting (upper panel) or activating (lower panel) NRP1 ligands oppositely modulates its pro-endocytic function. Inhibitory ligands, such as mini-WARS and other aaRSs, effectively binds the extracellular portion of NRP1, impair NRP1 endocytosis and stabilize its localization at AJs. Therefore, mini-WARS inhibits junctional VE-cadherin turnover and histamine-elicited paracellular endothelial permeability. On the contrary, activatory ligands, such as VEGF-A, SEMA3A and likely other aaRSs, increase the internalization of NRP1 and VE-cadherin from AJs.
