# Supplementary material for: Neuropilin 1 and its inhibitory ligand mini-tryptophanyl-tRNA synthetase inversely regulate VE-cadherin turnover and vascular permeability
Source: Nat Commun. 2022 Jul 20;13:4188. doi: 10.1038/s41467-022-31904-1 (PMC9300702; doi:10.1038/s41467-022-31904-1)
Supplement: Supplementary file 3 — Description of Additional Supplementary Files [file 41467_2022_31904_MOESM3_ESM.pdf]

Description of Additional Supplementary Files

File name: Supplementary Data 1

Description: MS proteomic data of NRP1 interactome.

File name: Supplementary Data 2

Description: KEGG pathway enrichment analysis performed with STRING.

File name: Supplementary Data 3

Description: Graph data of Supplementary Figure 3.
