# Supplementary material for: Neuropilin 1 and its inhibitory ligand mini-tryptophanyl-tRNA synthetase inversely regulate VE-cadherin turnover and vascular permeability
Source: Nat Commun. 2022 Jul 20;13:4188. doi: 10.1038/s41467-022-31904-1 (PMC9300702; doi:10.1038/s41467-022-31904-1)
Supplement: Supplementary file 7 — Reporting Summary [file 41467_2022_31904_MOESM7_ESM.pdf]

nature research

Corresponding author(s): Guido Serini and Sara Zanivan

Last updated by author(s): Jun 16, 2022

Reporting Summary

Nature Research wishes to improve the reproducibility of the work that we publish. This form provides structure for consistency and transparency in reporting. For further information on Nature Research policies, see our Editorial Policies and the Editorial Policy Checklist.

Statistics

For all statistical analyses, confirm that the following items are present in the figure legend, table legend, main text, or Methods section.

n/a	Confirmed	
☐	☒	The exact sample size (*n*) for each experimental group/condition, given as a discrete number and unit of measurement
☐	☒	A statement on whether measurements were taken from distinct samples or whether the same sample was measured repeatedly
☐	☒	The statistical test(s) used AND whether they are one- or two-sided<br>*Only common tests should be described solely by name; describe more complex techniques in the Methods section.*
☐	☒	A description of all covariates tested
☐	☒	A description of any assumptions or corrections, such as tests of normality and adjustment for multiple comparisons
☐	☒	A full description of the statistical parameters including central tendency (e.g. means) or other basic estimates (e.g. regression coefficient) AND variation (e.g. standard deviation) or associated estimates of uncertainty (e.g. confidence intervals)
☐	☒	For null hypothesis testing, the test statistic (e.g. *F*, *t*, *r*) with confidence intervals, effect sizes, degrees of freedom and *P* value noted<br>*Give P values as exact values whenever suitable.*
☒	☐	For Bayesian analysis, information on the choice of priors and Markov chain Monte Carlo settings
☐	☒	For hierarchical and complex designs, identification of the appropriate level for tests and full reporting of outcomes
☒	☐	Estimates of effect sizes (e.g. Cohen's *d*, Pearson's *r*), indicating how they were calculated

Our web collection on statistics for biologists contains articles on many of the points above.

Software and code

Policy information about availability of computer code

Data collection	The .raw MS file and search/identification files obtained with MaxQuant computational platform91 version 1.5.0.26. The source code and the executable that have been used to annotate the topology and subcellular location of NRP1 interactors can be found in my github repository https://github.com/gkoulouras/uniprot-annotation-downloader. For image quantification, Image ProPlus 6.2 software (Media Cybernetics) and Leica Las-X software.
Data analysis	For statistical evaluation, parametric two-tailed heteroscedastic Student's t test was used to assess the statistical significance when two groups of unpaired normally distributed values were compared; when more than two groups were compared, parametric two-tailed analysis of variance (ANOVA) with Bonferroni's post hoc analysis was applied. Statistical differences were considered not significant (ns) = p-value > 0.05; significant * = p-value ≤ 0.05; ** = p-value ≤ 0.01; *** = p-value ≤ 0.001). For MS analysis the proteinGroup.txt output of MaxQuant was analyzed with Perseus. In the Supplementary data we also reported the adjusted p-values (q-values), which were calculated using Benjamini-Hockbert FDR. The selection of transmembrane proteins from the NRP1 interactome was based on the UniProt32 "Topology" annotation. STRING50 was used for category enrichment analysis (KEGG110 functional annotation categories were used).

For manuscripts utilizing custom algorithms or software that are central to the research but not yet described in published literature, software must be made available to editors and reviewers. We strongly encourage code deposition in a community repository (e.g. GitHub). See the Nature Research guidelines for submitting code & software for further information.

Data

Policy information about availability of data

All manuscripts must include a data availability statement. This statement should provide the following information, where applicable:

- Accession codes, unique identifiers, or web links for publicly available datasets
- A list of figures that have associated raw data
- A description of any restrictions on data availability

The .raw MS files have been deposited to the ProteomeXchange Consortium (http://proteomecentral.proteomexchange.org/cgi/GetDataset) via the PRIDE partner repository113 with dataset identifier PXD019700 (login information for Reviewers: Username: reviewer06199@ebi.ac.uk; Password: ieIOXWKk). STRING was used for category enrichment analysis (KEGG functional annotation categories, which were reported in the Analysis output of STRING, were used)

Field-specific reporting

Please select the one below that is the best fit for your research. If you are not sure, read the appropriate sections before making your selection.

☒ Life sciences ☐ Behavioural & social sciences ☐ Ecological, evolutionary & environmental sciences

For a reference copy of the document with all sections, see nature.com/documents/nr-reporting-summary-flat.pdf

Life sciences study design

All studies must disclose on these points even when the disclosure is negative.

Sample size	For the Western Blot analysis, we used our historical and preliminary data to calculate that most of our experiments must be run in duplicate or more. For the adhesion assay, the same was done to calculate that our experiments were done in biological triplicates (3 technical replicates each).For the IF analysis, the same was done including 100 cells for condition pooled from 2 independent experiments. For the FRAP analysis, the same was done including 40 cells for condition pooled from 5 independent experiments. For the ELISA capture assay the same was done, in biological triplicates (3 technical replicates each). For the in vitro permeability assay, the same was done in biological triplicates (3 technical replicates each). For in vivo permeability the same was done, including 5 mice per group.
Data exclusions	In the MS proteomic data common reverse and contaminant hits (as defined in MaxQuant) were removed, as well as proteins only identified by site. The LFQ Intensity values were used for protein quantification. Only proteins quantified in at least three of the four biological replicates in at least one experimental group were kept for follow up analysis. LFQ values were log2 scaled and missing values were replaced using the Imputation function (set up: from normal distribution, width 0.3 and down shift 1.8). Proteins that had a fold increase of at least 1.5 compared to the control and p-value lower than 0.05 were considered potential NRP1 interactors.
Replication	For all the assays, the observed differences were confirmed over the four first experiments, performed over a month.
Randomization	For each protein, its enrichment in the NRP1 surface interactome and NRP1 endosomal interactome was calculated by dividing the LFQ intensity to the LFQ intensity measured in the control sample (Ctl = ECs transduced with empty pCCL lentivirus). Mice were grouped by genotype. For this reason there was no randomization necessary in this experiment.
Blinding	Investigators were blinded.

Reporting for specific materials, systems and methods

We require information from authors about some types of materials, experimental systems and methods used in many studies. Here, indicate whether each material, system or method listed is relevant to your study. If you are not sure if a list item applies to your research, read the appropriate section before selecting a response.

Materials & experimental systems

n/a	Involved in the study	
☐	☒	Antibodies
☐	☒	Eukaryotic cell lines
☒	☐	Palaeontology and archaeology
☐	☒	Animals and other organisms
☐	☒	Human research participants
☒	☐	Clinical data
☒	☐	Dual use research of concern

Methods

n/a	Involved in the study	
☒	☐	ChIP-seq
☒	☐	Flow cytometry
☒	☐	MRI-based neuroimaging

Antibodies

Antibodies used	Mouse monoclonal antibody (mAb) anti-HaloTag (Promega, G921A, for Western Blot -WB- 1:1000); mAb anti-α tubulin (Sigma-Aldrich, T5168/clone B-5-1-2, WB 1:8000); goat polyclonal anti-EEA1 (Santa Cruz Biotechnology, N-19/sc-6415, immunofluorescence-IF-1:200); rabbit anti VEGFR-2 (Cell Signaling, 55B11/#2479, WB 1:1000); mAb anti-NRP1 (R&D Systems, MAB3870, IF 1:50, live for immunoprecipitation-IP-1:2000); goat polyclonal Anti- VE-cadherin (Santa Cruz Biotechnology, C-19/sc-6458, IF 1:200, ELISA 1µg/ml); rabbit anti- VE-cadherin (Cell Signaling, D87F2, WB 1:1000); rabbit anti-NRP1 (Abcam, EPR3113, WB 1:3000); mAb anti-PECAM1 (Cell Signaling, 89C2/#3528, WB 1:1000, IF 1:50); goat polyclonal anti-NRP1 (Santa Cruz Biotechnology, C19/sc-7239, IP 1µg/ml lysate); rabbit polyclonal anti-WARS (Thermo Fisher Scientific, PA5-29102, WB 1:1000); mAb anti-vinculin (Sigma-Aldrich, V9131, WB 1:2000); rabbit anti-V5 (Cell Signaling, D3H8Q, IP 1µg/ml lysate)rat monoclonal Ab anti-HA (Roche, 11867423001, WB 1:1000); mAb anti-V5 (Santa Cruz Biotechnology, E10/sc-81594, WB 1:1000); mAb anti S100A10 (Santa Cruz Biotechnology, 4E7E10/sc-81153, WB 1:500); mAb anti-transferrin receptor 1 (Santa Cruz Biotechnology, CD71/G-8/sc-393719, ELISA 1µg/ml); polyclonal sheep anti-NRP1 (R&D Systems, AF3870, ELISA 1µg/ml); For mouse tissue immunofluorescence analyses, rabbit monoclonal Ab anti-Nrp1 (Abcam, ab81321, mouse tissue IF 1:100); rat monoclonal Ab anti-panendothelial cell antigen Meca32 (BD Pharmingen, 550563, mouse tissue IF 1:100); goat anti-rabbit secondary Ab (Jackson ImmunoResearch Laboratories, 111-035-003, WB 1:20000); goat anti-mouse secondary Ab (Jackson ImmunoResearch Laboratories, 115-035-003, WB 1:20000); goat anti-rat HRP (Abcam, ab97057, WB 1:2000); Alexa Fluor 488 donkey anti-mouse (A21202) and anti-goat (A11055) IgG (H+L) secondary Ab (Life Technologies, IF 1:400); Alexa Fluor 555 donkey anti-mouse (A31570) and anti-goat (A21432) IgG (H+L) secondary Ab (Life Technologies, IF 1:400); Alexa Fluor 647 donkey anti-mouse (A31571) and anti-goat (A21447) IgG (H+L) secondary Ab (Life Technologies, IF 1:400);DAPI (Life Technologies, D3571); normal Goat (NI02-100UG) and mouse (NI03-100UG) IgGs (Millipore, IP 1µg/ml lysate); mouse monoclonal agarose conjugated anti NRP1 antibody (Santa Cruz Biotechnology, A-12/sc-5307, 1µg pull down assay); goat anti-human IgG Fc HRP preadsorbed (Abcam, ab98624, WB 1:10000); agarose anti-V5 tag antibody (Abcam, ab1229, 0.2 µg/ml medium); mAb anti-GAPDH (Abcam, 6C5/ab8245, WB 1:10000).
Validation	We chose the best Antibodies for the species (human or mouse) and applications (Western Blot, Immunoprecipitation and Immunofluorescence) as indicated in the manufacturer's website.

Eukaryotic cell lines

Policy information about cell lines

Cell line source(s)	The isolation of primary arterial ECs from human umbilical cords was approved by the Office of the General Director and Ethics Committee of the Azienda Sanitaria Ospedaliera Ordine Mauriziano di Torino hospital (protocol approval no. 586, Oct 22 2012 and no. 26884, Aug 28 2014) and informed consent was obtained from each patient.
Authentication	Cells not authenticated as primary cells were used.
Mycoplasma contamination	Cells were tested for mycoplasma contamination by means of Venor GeM Mycoplasma Detection Kit (MP0025-1KT, Sigma Aldrich).
Commonly misidentified lines (See ICLAC register)	n/a

Animals and other organisms

Policy information about studies involving animals; ARRIVE guidelines recommended for reporting animal research

Laboratory animals	Mus musculus domesticus, C57Bl/6J, 12 animals, 8 females, 4 males. All wild type mice used in the experiment were aged 13 weeks. These animals were obtained from the KU Leuven Laboratory Animal Center (Leuven, Belgium). The following parameters are checked and noted daily:<br>Illumination: is controlled on a 14h light, 10h dark light cycle from 7h to 21h<br>Temperature is checked daily and should be 22±2°C<br>Humidity in mouse rooms is checked daily and should be between 45-70%.<br><br>Mus musculus domesticus, C57Bl/6J-Nrp1<tm2Ddg>-Tg(Cdh5-cre/ERT2)1Rha, 16 animals, 8 females, 8 males.<br>All mice were genotyped as being Nrp1 lox/lox and Cdh5-Cre +/- .<br>All knock-out mice used in the experiment were aged between 11 and 20 weeks.<br>The animals were obtained from the Laboratory of Transgenic Mouse Models, IRCCS, Candiolo, Italy.<br>Tg(Cdh5-cre/ERT2)1Rha (Ralf H Adams - MGI:3848982) is a tamoxifen-inducible cre/ERT2 sequence under control of the vascular endothelial cadherin (Cdh5(PAC)) promoter.<br>Nrp1<tm2Ddg> (David D Ginty - MGI:3512101) is a targeted conditional ready mutation of the neuropilin 1 gene in which loxP sites flank exon 2. When bred to a Cre-recombinase expressing strain, the Cre transgenic offspring is susceptible to tamoxifen induced recombination resulting in the tissue-specific deletion of the exon 2 and thus knock-out of the Nrp1.
Wild animals	No wild animals were used for this study.
Field-collected samples	No field-collected samples were used for this study.
Ethics oversight	Housing and all experimental animal procedures were approved by the Institutional Animal Care and Research Advisory Committee of the KU Leuven and by the ethics committee of the University of Torino and by the Italian Ministry of Health (Protocol approval no. 337/2016-PR).

Note that full information on the approval of the study protocol must also be provided in the manuscript.

Human research participants

Policy information about studies involving human research participants

Population characteristics	The primary Artherial ECs from umbilical cords were obtained from informed healthy pregnant women.
Recruitment	Informed consent was obtained from each patient.
Ethics oversight	The isolation of primary arterial ECs from human umbilical cords was approved by the Office of the General Director and Ethics Committee of the Azienda Sanitaria Ospedaliera Ordine Mauriziano di Torino hospital (protocol approval no. 586, Oct 22 2012 and no. 26884, Aug 28 2014).

Note that full information on the approval of the study protocol must also be provided in the manuscript.
