# Supplementary material for: Neuropilin 1 and its inhibitory ligand mini-tryptophanyl-tRNA synthetase inversely regulate VE-cadherin turnover and vascular permeability
Source: Nat Commun. 2022 Jul 20;13:4188. doi: 10.1038/s41467-022-31904-1 (PMC9300702; doi:10.1038/s41467-022-31904-1)
Supplement: Supplementary file 8 — Source Data [file 41467_2022_31904_MOESM8_ESM.zip › Gioelli et al_Source data 2.pdf]

Figure 1.

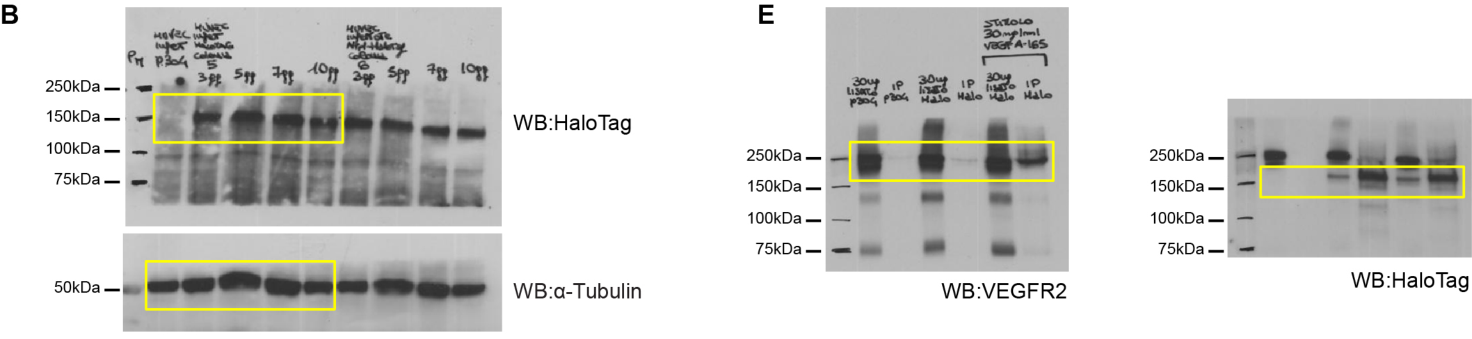

Figure 3.

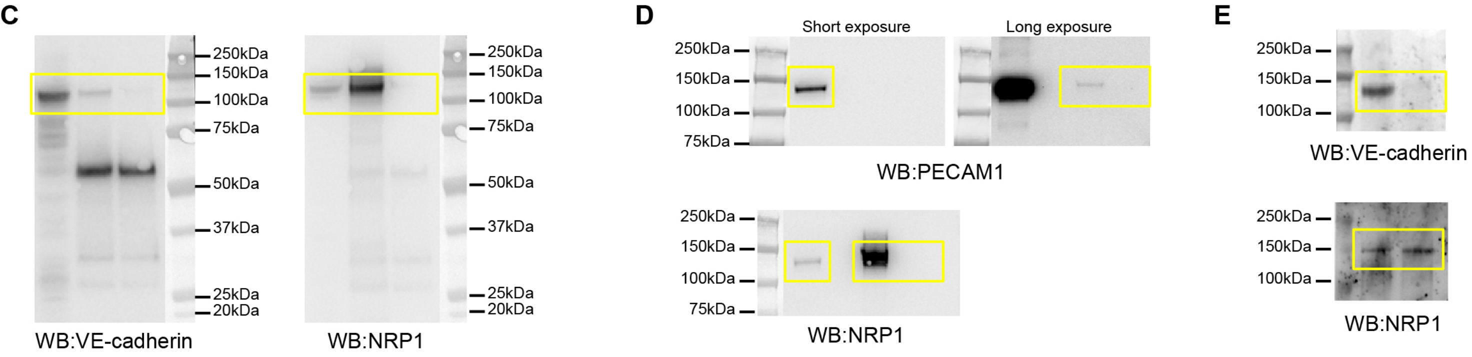

Figure 4.

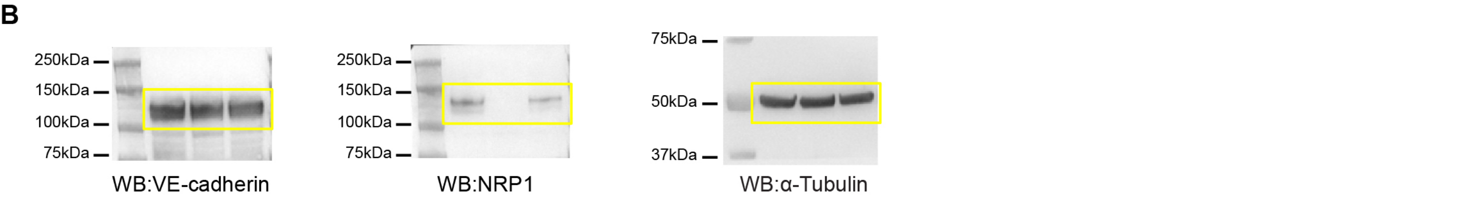

Figure 5.

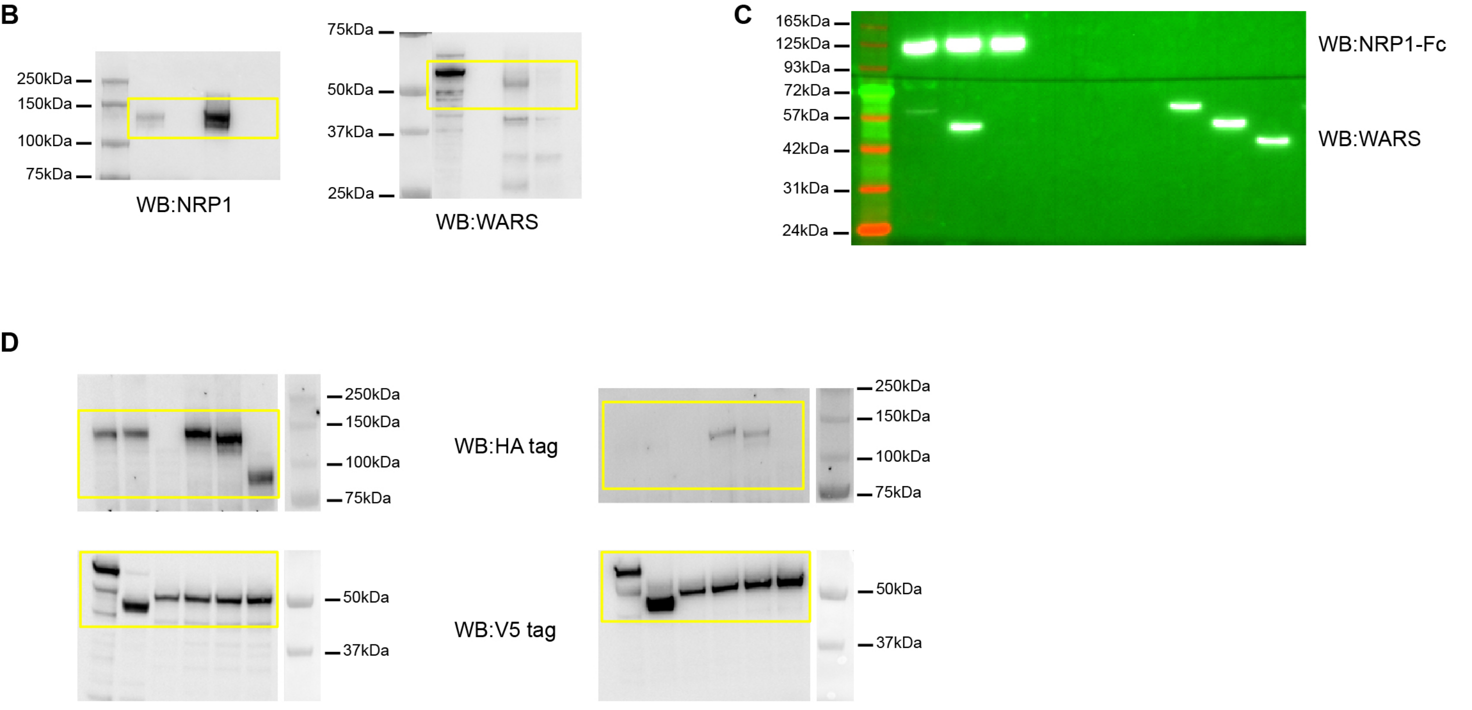

Supplementary Data 4.
Full images of blots that were cropped in main Figure 1,3,4 and 5. Yellow squares indicate the cropped images.

Figure 5.

E

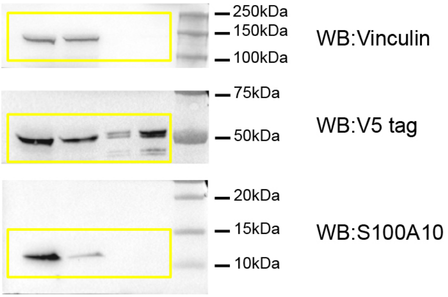

G

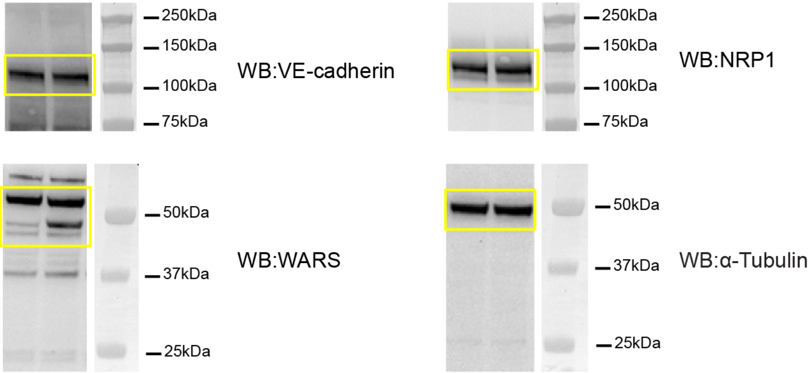

Figure 6.

A

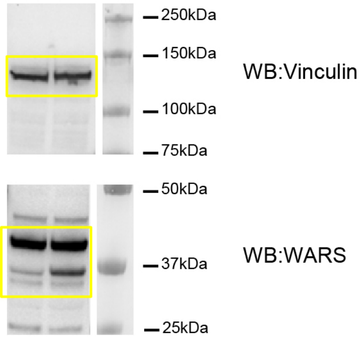

B

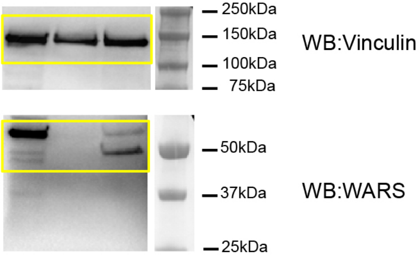

C

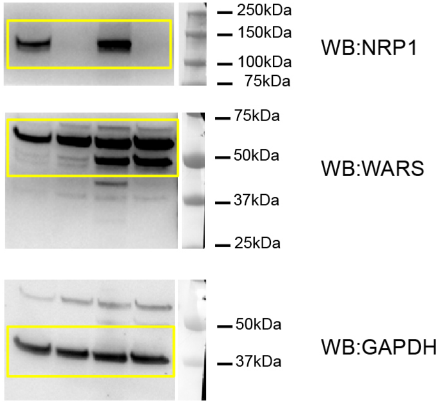

Supplementary Data 4.

Full images of blots that were cropped in main Figure 5 and 6. Yellow squares indicate the cropped images.
